# Differences in Physical Match Performance and Injury Occurrence Before and After the COVID-19 Break in Professional European Soccer Leagues: A Systematic Review

**DOI:** 10.1186/s40798-022-00505-z

**Published:** 2022-09-30

**Authors:** Maximiliane Thron, Peter Düking, Sascha Härtel, Alexander Woll, Stefan Altmann

**Affiliations:** 1 Department for Performance Analysis, Institute of Sports and Sports Science, Karlsruhe Institute of Technology, Engler-Bunte-Ring 15, 76131 Karlsruhe, Germany; 2 Integrative and Experimental Exercise Science and Training, Institute of Sport Science, University of Würzburg, Würzburg, Germany; 3 TSG, 1899 Hoffenheim, Zuzenhausen, Germany; 4 Department for Social and Health Sciences in Sport, Institute of Sports and Sports Science, Karlsruhe Institute of Technology, Karlsruhe, Germany; 5 TSG ResearchLab gGmbH, Zuzenhausen, Germany

**Keywords:** Football, Pandemic, Match analysis, High-intensity, Sprinting

## Abstract

**Background:**

Due to the COVID-19 pandemic, matches and soccer-specific training were suspended for several weeks, matches after resumption were congested, and substitutions per team and game increased from three to five.

**Objective:**

The aim of this review was to examine possible differences in physical match performance and injuries between before and after the COVID-19 induced break of matches and training in professional male European soccer leagues during the 2019/2020 season.

**Methods:**

A systematic search identified all scientifically peer-reviewed publications involving elite male soccer players competing in the European leagues which reported physical match performance variables such as total running distance and running distance at different speed zones and/or injury parameters pre- and post-COVID-19 induced break.

**Results:**

In total, 11 articles were included, which were coming from German Bundesliga, Polish Ekstraklasa, Croatian HNL, Spanish La Liga, and Italian Serie A. In all studies investigating the German Bundesliga, most parameters of physical match performance remained unaffected (0.08 ≤ *p* ≤ 0.82; − 0.15 ≤ ES 0.15), while studies investigating the Polish Ekstraklasa (*p* ≤ 0.03; − 0.27 ≤ ES − 0.18), Croatian HNL (*p* ≤ 0.04; − 1.42 ≤ ES ≤ 1.44), Spanish La Liga (*p* ≤ 0.017; − 0.32 ≤ ES ≤ 5.5), and Italian Serie A (*p* ≤ 0.014; − 1.01 ≤ ES 0.24) showed a decrease in most parameters of physical match performance after the COVID-19 break. Injury rates were only investigated by studies targeting the German Bundesliga and Italian Serie A. In the majority of studies (3 out of 4 studies), there occurred no difference in injuries between pre- and post-COVID-19 break (*p* > 0.05; ES = N/A).

**Conclusion:**

Results indicate that Bundesliga teams maintained physical match performance during the 9-weeks break in matches and 3-weeks break in group training, whereas a longer match and group training interruption up to 15 weeks and 8 weeks, respectively, in the other leagues appeared to lead to a decreased physical match performance. Regarding injuries, we speculate that the increase in substitutions from 3 to 5 substitutions per game might prevent an increase in injury occurrence during matches. The underlying studies’ results provide hints for possible upcoming unexpected interruptions with respect to optimal physical preparations for the resumption of matches and a congested schedule to maintain physical match performance, or for possible rule changes such as 5 instead of 3 substitutions to avoid physical overload during congested match schedules.

**Supplementary Information:**

The online version contains supplementary material available at 10.1186/s40798-022-00505-z.

## Key Points

1. Due to the COVID-19 pandemic, matches and soccer-specific training in professional European soccer were suspended for several weeks with differences in durations between countries, and congested matches after resumption.
2. German Bundesliga teams seemed to maintain physical match performance during the 9-weeks break in matches and 3-weeks break in group training, whereas a longer match and group training interruption up to 15 weeks and 8 weeks, respectively, in Polish Ekstraklasa, Croatian HNL, Spanish La Liga, and Italian Serie A appeared to lead to a decreased physical match performance.
3. In most studies, there occurred no differences in injuries between pre to post COVID-19 break. The temporary rule change from 3 to 5 substitutions per game might prevent an increase in the occurrence of injuries during matches.

## Introduction

The COVID-19 pandemic heavily impacted professional soccer worldwide during season 2019/2020. In Europe, most leagues suspended matches and team training between March and June 2020 to contain the spread of the virus. Teams had to stop their regular training for several weeks and were only allowed to conduct individual "home-based training", i.e., individualized endurance and strength training at home to reduce detraining effects [1, 2]. The restart of group training took place after several weeks in small groups whereby the duration of breaks differed between the leagues. For example, German Bundesliga had the shortest interruption of matches with 9 weeks and restarted group training after 3 weeks [3], while Italian Serie A suspended matches for 15 weeks and group training for 8 weeks [4]. Additionally, after resumption of matches, no spectators were allowed into the stadiums and match schedules were congested. Matches per week increased from 0.9–1.0 to 1.5–2.0 from pre to post COVID-19 induced break to end season 2019/2020 “in time” following the COVID-19 break [3, 4]. In order to account for the limited soccer-specific preparation time and to avoid physical overload of the individual players during the congested match schedule, the number of possible substitutions per match was elevated from three to five [5].

Thus, the COVID-19 pandemic affected soccer match operations in season 2019/2020 in a unique and incomparable way to previous seasons and with differences between European leagues regarding the duration of the break in matches and soccer-specific training and match frequency after the resumption. Research on possible detraining effects during the COVID-19 break in matches and soccer-specific training indicate reduced performance in sprint and jump tests which can be associated with the decrease in soccer-specific (group) training during lockdown [6, 7]. Regarding congested match schedules, research unrelated to the COVID-19 situation points out a potential increasing impact on injury occurrence and decreasing effect on physical match performance due to congested match schedules [8–11]. Numerous studies examined physical match performance and injury numbers before and after the Covid-19 induced break in matches and training in different European professional male soccer leagues [3, 4]. However, the topic is currently not systematically reviewed.

Therefore, the aim here was to systematically review the scientific literature regarding differences in physical match performance and injuries between pre- and post-COVID-19 induced break of matches and training in professional male European soccer leagues. The results of the review can help to understand how the European leagues responded to the COVID-19 induced break in matches and training alongside different match operations after the break, and how best to act in case a similar situation occurs again.

## Methods

This systematic review was written according to the PRISMA (Preferred Reporting Items for Systematic Reviews and Meta-Analyses; see PRISMA 2020 checklist in Additional file 1: Supplementary Material A) guidelines [12]. The protocol was not registered before the start of the project.

### Inclusion Criteria

This review sought to identify all scientifically peer-reviewed publications involving elite male soccer players competing in the European leagues which reported physical match performance and/or injury parameters pre- and post-COVID-19 induced break.

#### Study Populations

All articles reporting on healthy elite adult male soccer players active in the first national European leagues were included.

#### Interventions

Studies were included if they specifically evaluated a pre vs. post COVID-19 break comparison on parameters of physical match performance and/or injury parameters in elite soccer players competing in the European leagues.

#### Outcomes Examined

Assessment of physical match performance parameters (e.g., total distance, running distance at different speeds, sprinting distances, counts of accelerations, and decelerations) and injury related parameters (e.g., injury incidence/rate and injury occurrence for injury types such as injuries of muscles, brain, tendons, ligaments, or bones) for pre- and post-COVID-19 induced break was conducted.

#### Publication Status and Language

**Fig. 1:**
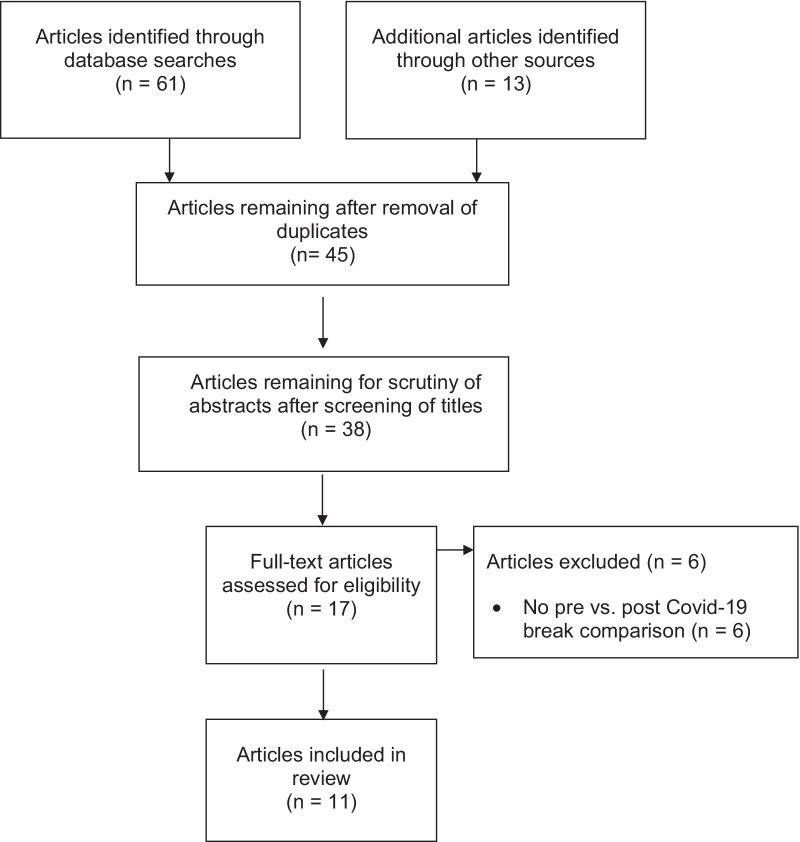
Selection of the articles to be analyzed, from initial identification to inclusion (Page et al. [12])

Our search was limited to original articles published in peer-reviewed journals and written in English. References cited by the articles retrieved were also examined for potential relevance. Conference abstracts, dissertations, theses, and other non-peer-reviewed articles were excluded. Figure 1 illustrates the screening and selection process employed.

### Search Strategy

A comprehensive search strategy was designed by the authors of this article and checked by a librarian to identify relevant studies. The electronic databases searched in December 2021 included PubMed and Web of Science (with no restriction concerning publication date) with an updated search being conducted in February 2022. The search strategy is illustrated using the search terms entered into the PubMed database as an example (Additional file 1: Supplementary Material C) and was modified according to the indexing systems of Web of Science. In the PubMed database, the build-in “COVID-19” filter was applied.

### Selection of Studies

The identified articles were incorporated into EndNote X9 (Clarivate Analytics, Philadelphia, USA), where duplicates were eliminated. The titles and abstracts of all potentially relevant articles were screened for eligibility by one of the authors (MT), with independent verification by a second author (PD). The full texts of articles that met the criteria for inclusion were then retrieved and screened. When disagreements between reviewers arose, consensus was achieved through discussion or input from a third author (SA).

### Data Extraction and Analysis

From the selected articles, one author (MT) extracted data which were independently confirmed by another (SA). Extracted information concerned: details of publication (authors, year, journal, publication date), characteristics of the study sample (league, match days, included seasonal periods), general conditions during different seasonal periods (duration of COVID-19 induced break, substitutions allowed, spectators allowed, and congestion of matches), and outcomes (absolute values and/or mean differences in physical match performance and/or injury parameters pre- and post-COVID-19 related lockdown).

To detect possible pre vs. post lockdown differences, p-values and effect sizes (ES) were recorded. ES were rated according to Cohen [13]: less than 0.2 was considered a trivial effect, 0.2 ≤ ES < 0.5 small effect, 0.5 ≤ ES < 0.8 moderate effect, and ES ≥ 0.8 large effect.

### Assessment of Methodological Quality

As recommended [14], we assessed methodological quality of the included articles using the National Institute of Health quality assessment tool for before-after (Pre-Post) study without control group (described in detail elsewhere: [15]. In brief, internal validity of a study is judged by 12 questions (e.g., sufficient sample size, clear study objective, validity, and reliability of assessment tools). The risk of bias was assessed independently by two of the authors (PD & MT), with any disagreements again being resolved by consensus or through discussion with a third author (SA). Mean kappa agreement between the authors was 0.90 (almost perfect agreement [16]).

## Results

### Study Characteristics

Of the 74 articles initially identified, only 11 fulfilled all the criteria for inclusion in the analysis and were published in 2021 or 2022. One article [17] investigated two different leagues. Thus, we included 11 articles, but 12 studies with investigations about different leagues.

The study characteristics, including number of match days, league, general match conditions, and variables are summarized in Table 1.

**Table 1:** Overview of characteristics of leagues and results of studies included

League (country) Duration of break (restart of group training)	General conditions (pre vs. post)	Study	N	Variables (definition)
*Bundesliga (Germany)* 9 weeks (after 3 weeks)	Substitutions: 3 vs. 5 Spectators: yes vs. no Matches/week: 1.0 vs. 1.5	Krutsch et al. [23]	26 match days (pre1 = first 8 match days of season 19/20, pre2 = 9 match days before break, post = 9 match days after break)	Injury incidence (fractures, joint, ligament, muscle, and tendon injuries, contusions, injuries of nervous system; injuries per 1000 football hours of training, matches, and overall exposure) [number/1000 h]
		Radziminski et al. [17]	34 match days (pre = 25 match days before break, post = 9 match days after break)	Total distance (sum of running distance of all players of a team) [m] Very high-intensity running (team distance at 21–24 km/h) [m] Sprinting (team distance > 24 km/h) [m]
		Santana et al. [18]	34 match days (pre = 25 match days before break, post = 9 match days after break)	Total distance (sum of running distance of all players of a team) [km] Sprints (team number of sprints with min. 1 s ≥ 22.68 km/h) [number]
		Seshadri et al. [24]	34 match days (pre = 25 match days before break, post = 9 match days after break)	Injury rate (any trauma or musculoskeletal injury that caused loss of game time) [team number/match]
		Thron et al. [3]	34 match days (pre = 25 match days before break, post = 9 match days after break)	Total distance (sum of running distance of all players of a team) [km] Average peak velocity (mean of maximum velocities in a team) [km/h] High-intensity distance (team distances at 17–23.99 km/h) [km] Sprinting distance (team distances ≥ 24 km/h) [km] Injury occurrence ((neuro-)muscular injuries, brain injuries, injuries to tendons, ligaments, or bones) [average injuries/team/match]
*Ekstraklasa (Poland)* 12 weeks (after 8 weeks)	Substitutions: 3 vs. 5 Spectators: yes vs. no Matches/week: 0.9 vs. 1.5	Radziminski et al. [17]	37 match days (pre = 26 match days pre, post = 11 match days after break)	Total distance (sum of running distance of all players of a team) [m] High-intensity running (team distance at 19.8–25.2 km/h) [m/min] Sprinting (team distance > 25.2 km/h) [m/min] High-intensity actions (team number of high-intensity runs and sprints) [number]
*Hrvatski Telekom PRVA Liga (Croatia)* 12 weeks (after 6 weeks)	Substitutions: 3 vs. 5 Spectators: yes vs. no Matches/week: 0.9 vs. 1.4	Sekulic et al. [20]	17 match days (pre = 7 match days after winter break and before Covid-19 break, post = 10 match days after break; 21 players from same team)	Position classification (central defenders, fullbacks, midfielders, forwards) Total distance (average of running distance of all players for the same position in a team) [m] Low-intensity running (mean player distance ≤ 14.3 km/h) [m] Running (mean player distance at 14.4–19.7 km/h) [m] High-intensity running (mean player distance ≥ 19.8 km/h) [m] Total accelerations (> 0.5 m/s^2^) [number] Total decelerations (< − 0.5 m/s^2^) [number]
*La Liga (Spain)* 13 weeks (after 8 weeks)	Substitutions: 3 vs. 5 Spectators: yes vs. no Matches/week: 1.0 vs. 1.9	Garcia-Aliaga et al. [19]	22 match days (pre = first 11 match days of season 19/20, post = 11 match days after break)	Distance (average per team per match) [km] Distance (distance covered per minute per team and per match) [m/min] Medium-speed running distance (team distance at 14.1–21 km/h) [m and m/min] High-speed running distance (team distance at 21.1–24 km/h) [m/min] Sprinting-speed running distance (team distance > 24 km/h) [m and m/min and number] Accelerations (team number > 3 m/s^2^) [number and number/min] Decelerations (team number ≤ 3 m/s^2^) [number and number/min] Maximum speed (maximum team speed) [km/h] Average speed (mean team speed) [km/h]
		Raya-González et al. [21]	38 match days (pre = 27 match days before break, post = 11 match days after break)	Team grouping into worsened ranking after break [WRS] and improved ranking after break [IMP] Total running distance (distance covered per team and minute) [m/min] High-intensity running distance (team distance at 21–24 km/h per minute) [m/min] Very high-intensity distance (team distance > 24 km/h per minute) [m/min] Accelerations (team number > 3 m/s^2^) [number] Decelerations (team number ≤ 3 m/s^2^) [number]
		De Souza et al. [22]	12 match days (pre = last match day before break, post = 11 match days after break)	Total running distance (sum of running distance of all players of a team) [km] Running distance < 14.0 km/h (team distance) [km] Running distance at 14.0–20.9 km/h (team distance) [km] Running distance at 21.0–23.9 km/h (team distance) [km] Running distance > 24.0 km/h (team distance) [km] Running actions > 24.0 km/h (sum of team) [number]
*Serie A (Italy)* 15 weeks (after 8 weeks)	Substitutions: 3 vs. 5 Spectators: yes vs. no Matches/week: 0.9 vs. 2.0	Rampinini et al. [7]	38 match days (pre1 = 12 match days until winter break; pre2 = 13 match days from winter break until lockdown; post = 13 match days after lockdown)	Total distance (mean of total match distances) [m/min] Very high-speed distance (mean match distances > 20 km/h) [m/min] Sprinting (mean match distances > 25 km/h) [m/min] High-acceleration distance (mean match distances > 3 m/s^2^) [m/min] High-deceleration distance (mean match distances < -3 m/s^2^) [m/min]
		Marotta et al. [25]	38 match days (pre = 26 match days before break, post = 12 match days after break)	Incidence rate (any injuries) [number of injuries × 1000) / (athletes in-game (at risk) × n° games × 1.5 (match hour)]

N—sample size

The studies included 364 match days in total (pre = 240 match days; post = 124 match days) involving the German Bundesliga (*n* = 162), Polish Ekstraklasa (*n* = 37), Croatian HNL (*n* = 17), Spanish La Liga (*n* = 72), and Italian Serie A (*n* = 76). Two studies which investigated German Bundesliga assessed only injuries, 2 studies only physical match performance, and one study assessed both. One study which investigated Polish Ekstraklasa examined only physical match performance, as well as one study for Croatian HNL and the 3 studies regarding Spanish La Liga. Of the 2 studies which investigated Italian Serie A, one examined physical match performance and the other injuries. Regarding physical match performance variables, total distance was included by 7 out of 12 studies, low-intensity running by 2 out of 12 studies, medium-intensity running by 3 out of 12 studies, high-intensity running by 7 out of 12 studies, sprinting distance by 8 out of 12 studies, acceleration parameters by 4 out of 12 studies, deceleration parameters by 4 out of 12 studies, and other parameters by 3 out of 12 studies. However, the definitions of the speed zones (i.e., low-intensity running, medium-intensity running, high-intensity running, and sprinting) differ between and partly within leagues, making direct comparisons difficult.

### Assessment of Methodological Quality

In all articles subject characteristics and outcomes were clearly presented and appropriate statistical methods were used (Additional file 1: Supplementary Material B). For data collection, 7 out of 8 articles used valid and reliable methods regarding physical match performance. Conversely, validity for data collection of injuries was not stated in all studies investigating injuries.

### General Conditions Pre vs. Post COVID-19 Break

The COVID-19 induced break in matches in German Bundesliga lasted 9 weeks, in Polish Ekstraklasa and Croatian HNL 12 weeks, in Spanish La Liga 13 weeks, and in Italian Serie A 15 weeks. Group training in Bundesliga was suspended for 3 weeks, in HNL for 6 weeks, and in Ekstraklasa, La Liga, and Serie A for 8 weeks. In all included European leagues, allowed substitutions increased from 3 to 5 per team and match. Spectators in soccer stadiums were allowed before, but not after the COVID-19 break in any herein analyzed league. The frequency of matches per week in all leagues before the interruption was 0.9–1.0 matches per week; after the break, the frequency in German Bundesliga and Polish Ekstraklasa was 1.5 matches per week, in Croatian HNL 1.4 matches per week, in Spanish La Liga 1.9 matches per week, and in Italian Serie A 2.0 matches per week.

### Physical Match Performance

Studies’ results regarding the pre vs. post COVID-19 break comparison of physical match performance and injuries are presented in Table 2.

**Table 2:** Studies’ results on selected parameters of physical match performance and injuries

Study	Physical Match Performance								Injuries
	Total distance	Low-intensity running (~ < 14 km/h)	Medium-intensity running (~ 14–20 km/h)	High-intensity running (~ 20–24 km/h)	Sprinting (~ > 24 km/h)	Accelerations	Decelerations	Others	
*Bundesliga (Germany)*									
Krutsch et al. [23]									Pre1: 5.4 injuries/1000 h MD: − 0.5 injuries/1000 h Pre2: 5.5 injuries/1000 h MD: − 0.6 injuries/1000 h Post: 4.9 injuries/1000 h *p* = 0.29 ES = N/A
Radziminski et al. [17]	Pre: 115 501.01 ± 4444.2 m Post: 114 822.2 ± 4530.9 MD: -678.81 m *p* = 0.08 ES = − 0.15			Pre: 3946.7 ± 480.1 m Post: 3961.7 ± 442.6 m MD: 15.00 m *p* = 0.70 ES = 0.03	Pre: 3074.4 ± 555.0 Post: 3110.5 ± 564.2 m MD: 36.10 m *p* = 0.20 ES = 0.06				
Santana et al. [18]	Pre: 116.18 ± 4.39 km Post: 115.26 ± 4.53 km MD: -0,92 km *p* < 0.05 ES = − 0.21				Pre: 220.45 ± 31.4 sprints Post: 446.2 ± 29.65 sprints MD: 2.65 sprints *p* = 0.45 ES = 0.09				
Seshadri et al. [24]									Pre: 0.27 injuries/match Post: 0.84 injuries/match MD: 0.57 injuries/match *p* < 0.001 ES = N/A
Thron et al. [3]	Pre: 115.50 ± 4.44 km Post: 114.82 ± 4.53 km MD: − 0.68 km *p* = 0.11 ES = 0.15			Pre: 10.95 ± 1.25 km Post: 11.01 ± 1.20 km MD: 0.06 km *p* = 0.61 ES = 0.05	Pre: 4.34 ± 0.73 km Post: 4.36 ± 0.74 km MD: 0.02 km *p* = 0.82 ES = 0.02			*Average peak velocity:* Pre: 33.62 ± 0.89 km/h Post: 33.90 ± 1.04 km/h MD: 0.28 km/h *p* < 0.01 ES = 0.30	Pre: 0.29 ± 0.55 injuries/team/match Post: 0.28 ± 0.50 injuries/team/match MD: − 0.01 injuries/team/match *p* = N/A ES = N/A
*Ekstraklasa (Poland)*									
Radziminski et al. [17]	Pre: 112 895.0 ± 4218.6 m Post: 111 714.4 ± 4614.1 m MD: -1180.6 m *p* < 0.01 ES = − 0.27			Pre: 7107.3 ± 838.5 m Post: 6961.2 ± 786.6 m MD: -146.10 m *p* = 0.03 ES = − 0.18	Pre: 1752.9 ± 357.7 m Post: 1720.6 ± 332.6 sprints MD: -32.30 m *p* > 0.05 ES = − 0.09			*High-intensity actions:* Pre: 602.3 ± 62.5 actions Post: 590.5 ± 60.3 actions MD: -11.80 actions *p* = 0.02 ES = − 0.20	
*Hrvatski Telekom PRVA Liga (Croatia)*									
Sekulic et al. [29]	*Central defenders* Pre: 10 247 ± 643 m Post: 10 211 ± 541 m MD: -36 m *p* = 0.85 ES = 0.06 *Midfielders* Pre: 11 492 ± 478 m Post: 11 668 ± 625 m MD: 176 m *p* = 0.33 ES = − 0.30 *Fullbacks* Pre: 11 106 ± 430 m Post: 10 549 ± 668 m MD: -557 m *p* = 0.06 ES = 0.98 *Forwards* Pre: 10 670 ± 605 m Post: 10 392 ± 912 m MD: -278 m *p* = 0.55 ES = 0.33	*Central defenders* Pre: 8323 ± 439 m Post: 8602 ± 486 m MD: 279 m *p* = 0.07 ES = − 0.62 *Midfielders* Pre: 8640 ± 318 m Post: 9063 ± 416 m MD: 423 m *p* = 0.01 ES = − 1.07 *Fullbacks* Pre: 8629 ± 404 m Post: 8316 ± 439 m MD: 47 m *p* = 0.81 ES = − 0.11 *Forwards* Pre: 7968 ± 274 m Post: 7875 ± 506 m MD: -93 m *p* = 0.71 ES = 0.20	*Central defenders* Pre: 1353 ± 256 m Post: 1157 ± 161 m MD: -196 m *p* = 0.01 ES = 0.94 *Midfielders* Pre: 2116 ± 207 m Post: 1937 ± 352 m MD: -179 m *p* = 0.07 ES = 0.57 *Fullbacks* Pre: 1819 ± 259 m Post: 1442 ± 220 m MD: -377 m *p* = 0.01 ES = 1.65 *Forwards* Pre: 1651 ± 270 m Post: 1703 ± 460 m MD: 52 m *p* = 0.82 ES = − 0.12	*Central defenders* Pre: 571 ± 217 m Post: 453 ± 130 m MD: -118 m *p* = 0.04 ES = 0.68 *Midfielders* Pre: 735 ± 197 m Post: 669 ± 181 m MD: -66 m *p* = 0.25 ES = 0.35 *Fullbacks* Pre: 1018 ± 162 m Post: 790 ± 160 m MD: -228 m *p* = 0.01 ES = − 1.42 *Forwards* Pre: 1051 ± 98 m Post: 814 ± 185 m MD: -237 m *p* = 0.02 ES = 1.42		*Central defenders* Pre: 475 ± 55 accelerations Post: 473 ± 55 accelerations MD: -2 accelerations *p* = 0.95 ES = 0.02 *Midfielders* Pre: 517 ± 34 accelerations Post: 490 ± 39 accelerations MD: -27 accelerations *p* = 0.02 ES = 0.71 *Fullbacks* Pre: 491 ± 44 accelerations Post: 466 ± 46 accelerations MD: -25 accelerations *p* = 0.24 ES = 0.58 *Forwards* Pre: 451 ± 45 accelerations Post: 445 ± 47 accelerations MD: -6 accelerations *p* = 0.80 ES = 0.14	*Central defenders* Pre: 471 ± 54 decelerations Post: 474 ± 54 decelerations MD: 3 decelerations *p* = 0.90 ES = − 0.04 *Midfielders* Pre: 512 ± 32 decelerations Post: 486 ± 38 decelerations MD: -26 decelerations *p* = 0.02 ES = 0.73 *Fullbacks* Pre: 481 ± 41 decelerations Post: 457 ± 42 decelerations MD: -24 decelerations *p* = 0.22 ES = 0.60 *Forwards* Pre: 441 ± 52 decelerations Post: 444 ± 47 decelerations MD: 3 decelerations *p* = 0.92 ES = − 0.06		
*La Liga (Spain)*									
Garcia-Aliaga et al. [19]	Pre: 75.62 ± 0.58 km Post: 70.20 ± 1.27 km MD: -5.42 km *p* < 0.001 ES = 5.5 Pre: 7699.22 ± 199.63 m/min Post: 7037.74 ± 216.19 m/min MD: -661.48 m/min *p* < 0.001 ES = 3.18		Pre: 104.42 ± 2.12 m Post: 103.59 ± 2.70 m MD: − 0.83 m *p* = 0.165 ES = 0.34 Pre: 202.91 ± 19.80 m/min Post: 186.67 ± 22.12 m/min MD: -16,24 m/min *p* = 0.001 ES = 0.77	Pre: 36.36 ± 2.40 m/min Post: 33.58 ± 2.51 m/min MD: − 2.78 m/min *p* < 0.001 ES = 1.13	Pre: 418.44 ± 28.33 m Post: 382.65 ± 32.83 m MD: -35.79 m *p* < 0.001 ES = 1.1 Pre: 24.89 ± 1.48 counts Post: 23.06 ± 1.59 counts MD: − 1.83 counts *p* < 0.001 ES = 1.19) Pre: 6.06 ± 0.41 m/min Post: 6.16 ± 0.51 m/min MD: 0.10 m/min *p* = 0.308 ES = 0.27	Pre: 47.07 ± 2.52 accelerations Post: 44.62 ± 2.53 accelerations MD: − 2.45 accelerations *p* < 0.001 ES = 0.97 Pre: 0.66 ± 0.03 accelerations/min Post: 0.68 ± 0.04 accelerations/min MD: 0.02 accelerations/min *p* = 0.001 ES = 0.57	Pre: 52.76 ± 3.00 decelerations Post: 49.39 ± 3.47 decelerations MD: -3.37 decelerations p < 0.001 ES = 1.04 Pre: 0.74 ± 0.04 decelerations/min Post: 0.75 ± 0.05 decelerations/min MD: 0.01 decelerations/min *p* = 0.017 ES = 0.22	*Maximum speed* Pre: 29.91 ± 0.23 km/h Post: 29.81 ± 0.36 km/h MD: − 0.10 km/h *p* = 0.115 ES = 0.33 *Average speed* Pre: 6.27 ± 0.13 km/h Post: 6.22 ± 0.26 km/h MD: − 0.05 km/h *p* = 0.184 ES = 0.34	
Raya-González et al. [21]	*WRS* Pre: 110.09 ± 0.37 m/min Post: 106.64 ± 0.18 m/min MD: -3.45 m/min *p* < 0.001 ES = -9.32 *IMP* Pre: 109.45 ± 0.44 m/min Post: 107.76 ± 0.25 m/min MD: − 1.69 m/min *p* < 0.001 ES = -3.84			*WRS* Pre: 3.93 ± 0.05 m/min Post: 3.73 ± 0.03 m/min MD: − 0.20 m/min *p* < 0.001 ES = -4.00 *IMP* Pre: 4.09 ± 0.03 m/min Post: 4.01 ± 0.04 MD: − 0.08 m/min *p* < 0.001 ES = − 2.67	*WRS* Pre: 2.96 ± 0.06 m/min Post: 2.75 ± 0.03 m/min MD: − 0.21 m/min *p* < 0.001 ES = -3.50 *IMP* Pre: 3.06 ± 0.06 m/min Post: 3.00 ± 0.03 m/min MD: − 0.06 m/min *p* > 0.05 ES = − 1.00	*WRS* Pre: 25.98 ± 0.06 accelerations Post: 26.11 ± 0.04 accelerations MD: + 0.13 accelerations *p* < 0.001 ES = 2.17 *IMP* Pre: 26.13 ± 0.08 accelerations Post: 26.00 ± 0.04 accelerations MD: − 0.13 accelerations *p* < 0.001 ES = − 1.63	*WRS* Pre: 25.83 ± 0.06 decelerations Post: 25.95 ± 0.04 decelerations MD: + 0.12 decelerations *p* < 0.001 ES = 2.00 *IMP* Pre: 25.97 ± 0.07 decelerations Post: 25.85 ± 0.04 decelerations MD: − 0.12 decelerations *p* < 0.001 ES = − 1.71		
De Souza et al. [22]	Pre: N/A Post: N/A MD: N/A *p* = n. s ES = N/A	Pre: N/A Post: N/A MD: N/A *p* = n. s ES = N/A	Pre: N/A Post: N/A MD: N/A *p* < 0.05 ES = N/A	Pre: N/A Post: N/A MD: N/A *p* = n. s ES = N/A	Pre: N/A Post: N/A MD: N/A *p* = n. s ES = N/A				
*Serie A (Italy)*									
Rampinini et al. [7]	Pre1: N/A Post: N/A MD: − 3.2 m·min − 1 *p* < 0.001 ES = − 0.58 Pre2: N/A Post: N/A MD: = − 5.4 m/min *p* < 0.001 ES = − 1.01			Pre1: N/A Post: N/A MD: − 0.8 m·min − 1 *p* < 0.001 ES = − 0.41 Pre2: N/A Post: N/A MD = − 1.0 m·min − 1 *p* < 0.001 ES = − 0.53	Pre1: N/A Pre2: N/A Post: N/A MD: N/A *p* = n. s ES = N/A	Pre1: N/A Post: N/A MD: N/A *p* = n. s ES = N/A Pre2: N/A Post: N/A MD = − 0.11 m/min *p* = 0.010 ES = 0.24	Pre1: N/A Post: N/A MD: N/A *p* = n. s ES = N/A Pre2: N/A Post: N/A MD: − 0.12 m/min *p* = 0.014 ES = − 0.23		
Marotta et al. [25]									Pre: N/A Post: N/A MD: v4 *p* > 0.05 ES = N/A

N/A—not available; p—significance level; MD—mean difference; ES—effect size; n. s.—not significant; WRS—teams that worsened ranking after break; IMP—teams that improved ranking after break

Physical match performance in German Bundesliga mainly did not change significantly from pre to post COVID-19 break in 3 out of 3 studies, i.e., high-intensity running distance (0.61 ≤ *p* ≤ 0.70; 0.03 ≤ ES 0.05) and sprinting distance (0.20 ≤ *p* ≤ 0.82; 0.02 ≤ ES ≤ 0.09) [3, 17, 18]. In 1 out of 1 study for Polish Ekstraklasa (*p* ≤ 0.03; − 0.27 ≤ ES ≤ − 0.18), in 1 out of 1 study for Croatian HNL (*p* ≤ 0.04; − 1.42 ≤ ES ≤ 1.44), in 2 out of 3 studies for Spanish La Liga (*p* ≤ 0.017; − 0.32 ≤ ES ≤ 5.5), and in 1 out of 1 study for Italian Serie A (*p* ≤ 0.014; − 1.01 ≤ ES ≤ 0.24), most physical match parameters such as total distance and high-intensity running distance decreased from pre to post [4, 17, 19–21]. 1 out of 3 studies for Spanish La Liga did not find significant differences in physical match performance, i.e., total distance, low-intensity running distance, high-intensity running distance, and sprinting distance (*p* = n. s.; ES = N/A), between pre- and post-COVID-19 break [22].

### Injuries

Krutsch et al. [23] as well as Thron et al. [3] did not find any differences in the German Bundesliga between pre- and post-COVID-19 break in injury incidence (*p* = 0.29; ES = N/A) and injury occurrence (*p* = N/A; ES = N/A), respectively. By contrast, Seshadri et al. [24] revealed a higher injury rate after the COVID-19 break compared to before in the Bundesliga (*p* < 0.001; ES = N/A). Injury rate in Serie A did not show remarkable differences in pre vs. post (*p* > 0.05; ES = N/A) [25].

## Discussion

The aim here was to systematically review the scientific literature regarding differences in physical match performance and injuries between pre- and post-COVID-19 induced break of matches and training of season 2019/2020 in professional European soccer leagues.

Most studies included in this review investigated the German Bundesliga (*n* = 5), followed by the Spanish La Liga (*n* = 3), Italian Serie A (*n* = 2), the HNL (*n* = 1) and Polish Ekstraklasa (*n* = 1).

The main findings of our review are:

1. In all studies (3 out of 3) investigating the German Bundesliga, parameters of physical match performance remained unaffected by the COVID-19 break
2. In the studies investigating the Polish Ekstraklasa (1 out of 1), Croatian HNL (1 out of 1), Spanish La Liga (2 out of 3), and Italian Serie A (1 out of 1), parameters of physical match performance decreased after the COVID-19 break
3. In the majority of studies (3 out of 4) investigating injury rates, there seems to be no difference from pre to post COVID-19 break in the German Bundesliga and Italian Serie A.

### Physical Match Performance

Although most physical match performance parameters in the German Bundesliga seemed to remain stable after the COVID-19 induced interruption of matches and training, some results differ between studies which could be due to different data collection techniques. Radzimiński et al. [17] and Thron et al. [3] used official match data provided by the “Deutsche Fussball Liga” which has been shown to be a valid and reliable data source [26, 27] and did not find differences in, e.g., total distance covered during match play, between pre- and post-COVID-19 break. Santana et al. [18] used data by the online-source Bundesliga.de and revealed decreasing total distance after the COVID-19 break. We could not find information regarding reliability and/or validity of Bundesliga.de.

We can only speculate on the reasons why there was no alteration in physical match performance in the German Bundesliga but in the other European leagues which showed a decrease in these parameters. One reason might be that the German Bundesliga teams stopped matches for 9 weeks and had only 3 weeks without group training, whereas Polish Ekstraklasa, Croatian HNL, Italian Serie A, and Spanish La Liga interrupted season 2019/2020 for 12–15 weeks without matches and restarted group training during that break after 6–8 weeks. Studies investigating physical and physiological capabilities in Italian and Colombian soccer players before and after the COVID-19 induced 15-weeks long interruption in matches and group training showed detraining trends in loss of lower body strength and power (− 15 to 17%), which can be associated with a decrease in soccer-specific (group) training during lockdown [7, 28]. The longer COVID-19 break in the Polish Ekstraklasa, Croatian HNL, Italian Serie A, and Spanish La Liga compared to the German Bundesliga potentially led to decreased physical and physiological capabilities which in turn negatively influenced physical match performance. Nevertheless, differences in home-based training between teams before the restart of group training are not considered due to lack of information. Moreover, results on physical match performance over different phases during a usual season indicate that, after the summer break, players need approximately 6–11 match days to stabilize their physical match performance [29]. However, the COVID-19 break differed from a conventional summer break as it appeared within the season, differed in duration, especially between leagues, and the amount of match days after the COVID-19 break was only as much as the match days needed to stabilize physical match performance after a summer break. With respect to the congested match schedules after the COVID-19 break, Bundesliga results in physical match performance support previous research which did not find a decline during congested match schedules [8, 9, 11, 30]. Conversely, in the Polish Ekstraklasa, Croatian HNL, Italian Serie A, and Spanish La Liga, congested match schedules could have resulted in the decline of physical match performance. The congestion of matches after the COVID-19 break was considerably higher in Spanish La Liga and Italian Serie A, with 1.9 and 2.0 games per week, respectively, compared to German Bundesliga, with 1.5 games per week, which could have led to the different results.

Several studies reported that the missing spectators during the match days after the COVID-19 break led to a reduced home-advantage or even a home-disadvantage [31, 32], which was potentially due to a reduced physical match performance of the home team. However, due to the complexity of different requirements and environmental conditions after the COVID-19 break, no clear conclusions about the interrelationships of the conditions and outcomes can be drawn.

In sum, the results indicate that Bundesliga teams maintained their physical match performance by using the opportunity to recover and work on physical weaknesses during the 9-weeks lockdown period, whereas a longer match and group training interruption up to 15 weeks and 8 weeks, respectively, in Ekstraklasa, HNL, Serie A, and La Liga appears to decrease physical match performance.

### Injuries

In most studies investigating injury rates, there seems to be no difference from pre to post COVID-19 break across all analyzed European soccer leagues (3 out of 4) [3, 23, 25]. These results contradict previous findings indicating an increase in injuries after an interruption of matches (i.e., winter break) and thus a short soccer-specific preparation time before the restart of matches [33] and during a congested match schedule [8, 10, 30].

Even though injury values in all studies of German Bundesliga are collected via transfermarkt.de (in case of Krutsch et al. [23] in combination with kicker magazine, Facebook, and Twitter), results of Krutsch et al. [23] and Thron et al. [3] disagree with those of Seshadri et al. [24]. It remains unclear why the results differ despite using the same database. However, no study has yet compared data from transfermarkt.de with those from established medical registries and therefore no clear statements can be made regarding validity. We can only speculate on the reasons why the injury rate did not change in the majority of studies, but it was previously argued that the increase of player substitution from 3 to 5 substitutions per game might reduce the risk of injury during matches by reducing the external load per player [34].

### Limitations

Our review included only a small number of studies and comparisons between soccer leagues are impaired due to an unequal number of studies available from each league. Furthermore, not every parameter was investigated in each league and parameter definition differed between studies which further impairs comparability. The data on injury occurrence are limited since, despite the articles included in our review drawing information mostly from official online-sources, it could be that not all teams report injuries truthfully to remain a competitive advantage and thereby injury results of the underlying studies should be treated with caution. Additionally, given the nature of the included articles, no control group was included in either study. Despite significant changes in some parameters of our review, we cannot confidently draw causal conclusions as to whether these changes where actually induced by the COVID-19 break or by possible confounding factors.

## Conclusions

We aimed to systematically review the scientific literature regarding differences in physical match performance and injuries between pre- and post-COVID-19 induced break of matches and training within season 2019/2020 in professional European soccer leagues.

We conclude that (i) the development of physical match performance from pre to post COVID-19 break differs between professional European soccer leagues; in all studies (3 out of 3) investigating the German Bundesliga, parameters of physical match performance remained unaffected by the COVID-19 break, (ii) in the studies investigating the Polish Ekstraklasa (1 out of 1), Croatian HNL (1 out of 1), Spanish La Liga (2 out of 3), and Italian Serie A (1 out of 1), parameters of physical match performance decreased after the COVID-19 break and (iii) in the majority of studies investigating injury rates, there occurred no difference from pre to post COVID-19 break in German Bundesliga and Italian Serie A (3 out of 4).

Although this is an unprecedented situation in soccer history, it cannot be ruled out that such a disruption may occur again. The underlying studies’ results indicate to teams and coaches the importance of physically preparing teams optimally for the resumption of matches and a congested schedule to maintain physical match performance, especially during longer interruptions. Although rule changes are rare in soccer, it seems reasonable to consider using five substitutions instead of three, to minimize physical overload effects from crowded schedules or, as expected in the future, from additional tournaments, and therefore reduce injury risk during matches.

## Supplementary Information

**Additional file 1.** **Supplementary Material A**. PRISMA 2020 checklist. **Supplementary Material B**. Assessment of methodological quality. **Supplementary Material C**. Search Terms Pubmed.

## Data Availability

Not applicable.
